# A data-driven short video international communication model based on indicator system communication network and attention BiLSTM neural network

**DOI:** 10.1038/s41598-024-65098-x

**Published:** 2024-06-24

**Authors:** Jinbao Song, Jing Liu

**Affiliations:** State Key Laboratory of Media Convergence and Communication, Communication University of China, Beijing, People’s Republic of China

**Keywords:** Engineering, Mathematics and computing

## Abstract

As one of the most popular ways to disseminate Internet content in the current era of media convergence, short videos have increasingly become part of people’s daily lives. This paper takes TikTok as the research object, focusing on the study of short video communication process. The research objective of this paper is to improve the international communication effect of short videos through data-driven analysis and strategy research on the law of international communication of short videos. Firstly, the key objective indicator system about international communication of short videos is constructed, the definition, selection and design logic of key objective indicators are introduced, the construction idea of the corresponding indicator system is proposed, and then the key objective indicator system about international communication of short videos and related data set are constructed. Secondly, an international communication network model for short videos is built. The construction of this network model is divided into three steps, the construction of the short video communication network KOIS-PN based on the key objective indicators system, the construction of AT-BiLSTM neural network model to adapt to the research scenario of short video international communication, the construction of an international communication network model of short videos combining KOIS-PN and AT-BiLSTM. The international short video communication network is constructed according to the key objective index system of international short video, and the key objective index system is used as nodes in the communication network, and the short video communication network is constructed by hierarchical relationship. Thirdly, the performance of the core theoretical model proposed in this paper is verified. The experimental results show that, the KOISPN-ATBiLSTM international communication network model of short videos has certain validity and advancement.

## Introduction

In the era of media convergence, social media has become an important place for people’s entertainment and recreation. As an important form of social media communication, short videos accompany people’s lives in various scenarios such as commuting, recreation, eating and before going to bed, and have become an important part of access to information, communication and even cognitive formation for people. Among several video APPs, TikTok, a strong international social media platform, has repeatedly surpassed Facebook, Instagram, Snapchat and YouTube in terms of downloads in the US domestic market^1^. SIR is a classical communication dynamics model, S is susceptible, I is infected and R is removed, a differential equation group based communication model is constructed, which and its derived models are actually based on the research of communication population. However, there are few studies on the communication models for short video communication, and the traditional communication models applied to short video communication research have certain limitations. For example, the social attributes of short videos are weak and the communication process is difficult to describe by classical communication models such as SIR and its derivative models^2^. Therefore, this study designs a method that combines the short video communication index system and neural network to explore the implicit relationship between different indexes and build the short video communication model.

## Related research

Among the communication-related studies of short videos, one major category is the short video communication studies based on humanities and social sciences, and another major category is the short video communication studies based on natural sciences^3^. Zhao^4^ attributed TikTok's success to the platform's recommendation algorithm. The study noted that TikTok's recommendation algorithm is jointly determined by video content and user behavior^5^, and the algorithm then feeds back to the videos and users, tagging them, and there is a connection between video tags and user tags. At the same time, TikTok has become a mainstream video media platform, the recommendation algorithm has been polished by billions of data, and the various data of the video is able to reflect the user characteristics and propagation laws accurately, which can provide a data base for the study of international communication of short video propagation.

In the field related to short video communication research based on humanities and social sciences, in Zhou^6^ based on the popular video content of Chinese culture theme released by folk subjects on TikTok platform, studied the current situation of international communication of Chinese culture in overseas social media platforms by the methods of case study and content analysis, made feature analysis of video content and analyzed the feedback effect of audience on video content in terms of awareness, emotion and behavior, and finally proposed targeted recommendations for the communication of Chinese culture on overseas social media platforms. In the same year, Zhang^7^ studied the influence of short video information dissemination in the new media era, taking the official accounts of news media organizations as research cases, crawled the user information data of short video news corresponding to the numbers of likes, comments, plays, pop-ups, set weights from three aspects, such as the click index, interaction index and utilization index, and constructed the short video communication influence index, measured the information dissemination influence of short video news by integrating multiple dimensions such as video broadcast length, video quantity, video content and production form, and analyzed the influence factors of its communication effect.

Among the studies related to short video communication based on natural science, Zhong^8^ modeled the popularity of online short videos on the TikTok platform, and the study pointed out that there are two typical communication patterns of single-gradient and multi-gradient for the cumulative plays of short videos, and constructed a like-communication dynamics model, and finally also analyzed the relationship between other factors and plays, which is a set of short video communication analysis methods based on communication dynamics. There are few algorithm studies directly for short video communication, but some scholars have made valuable studies in the field of communication research by applying the related algorithms involved in this study. Dang^9^ conducted a study on the algorithm of the social network information dissemination based on neural network, reconstructed the social network information dissemination model, solved the problem about constructing the state transfer equation of the traditional prediction model, optimized the social network information dissemination prediction by using algorithms in the field of neural network, proposed the algorithm of the social network information dissemination prediction based on LSTM, and established the framework of the social network information dissemination model based on LSTM to predict information dissemination process. The SEIR model is a mathematical model used to describe the spread of infectious diseases. It classifies populations into four categories: susceptible, exposed, infectious, and recovered. The SEIR model uses a set of differential equations to describe the dynamic transitions between these different categories. Liu^10^ applied deep learning methods to predict positive cases reported in Wuhan and four states in USA, and applied recurrent neural networks based on long-term and short-term memory and their bidirectional LSTM, stacked LSTM and traditional SEIR models to the Wuhan dataset to compare and select the best model for the positive case prediction task, and the results showed that the LSTM-based model performed significantly better than the traditional SEIR model.

This paper argues that for short video platforms, objective indicators can more accurately reflect some of the subjective factors that are difficult to specify. The short video platform must design recommendation algorithms with maximizing benefits of the platform as the starting point, such an algorithm must accurately grasp characteristics of the user in order to expand audiences and improve conversion, and the platform’s algorithms have been polished with billions of data, so these objective indicators are reliable enough to serve as nodes of the communication network and provide support for the research results.

At present, there are few related studies on the evaluation indicators of short videos at home and abroad. In the field of research on evaluation indicators of short videos, objective indicators and subjective indicators are usually fused to construct an indicator evaluation system. Yu^11^ constructed a government short video dissemination effect evaluation indicator system to provide a theoretical basis for the development of government short videos. This study used the Delphi method to conduct questionnaire surveys, scored the importance and availability of indicators, used the analytic hierarchy process to weight the government short video dissemination effect evaluation indicator system, constructed a government short video dissemination effect evaluation indicator system, and selected research samples to verify the feasibility, stability and persistence of the indicator system, and finally formulated strategies to improve the dissemination effect of government short videos. Nazarov^4^ conducted a study on TikTok network toolkits, determined the main factors affecting the content quality of personal accounts, established a fuzzy evaluation model to evaluate account content, and performed qualitative and quantitative analysis of the evaluation results. In the same year, Wang^12^ analyzed the first-level and second-level indicators of the short video network public opinion early warning indicator system under information epidemic by combining literature research with field research. Through a combination of analytic hierarchy and expert survey methods, they calculated the weights of various levels of indicators, and finally verified the scientificity and applicability of the information epidemic under short video network public opinion early warning indicator system constructed through empirical research.

While in the research of short video dissemination in reality, subjective indicators are difficult to verify their certainty, or the research work on determining subjective indicators is often large, and in this era of rapid change and high-speed development, the certainty of these subjective indicators changes over time. Therefore, the evaluation results of studies containing subjective indicators are also unclear and unstable. This paper believes that for short video platforms, objective indicators can more accurately reflect some difficult-to-clarify subjective factors, and short video platforms must design recommendation algorithms with maximizing platform benefits as the starting point, such algorithms must accurately grasp user characteristics in order to expand the audience and improve conversion, and the algorithm of the platform has been polished by hundreds of millions of data, so these objective indicators have enough reliability to serve as nodes of the communication network to provide support for research results. At the same time, the problem to be solved in this paper is how to improve the international dissemination effect of short videos, under this big problem, this paper separates its objective part which is to improve the international dissemination width of short videos, and conducts research on this problem.

RNN is a special internal neural network with self-connection, and the fields in which RNN and its derived models are applied are very numerous. Any problems that need to consider the temporal order and have time characteristics can be solved by using RNN and its derived models. The main drawback of RNN models is the problem of forgetting and vanishing gradient^13^. The methods to improve the forgetting problem of RNN models are^14^: (1) Changing the model structure of RNN into a more advanced model, such as LSTM, GRU, Bi-LSTM and so on; (2) combining other modules to overcome the forgetting problem, such as introducing attention mechanism. LSTM is the most common variant of RNN, and the core idea is that the concept of “gate” is introduced^15^. The problem solved by Bi-LSTM is that the unidirectional LSTM can only remember information before the current moment, and lacks grasp of information after the current moment. With Bi-LSTM, the model has the ability to save information in both directions of history and future. At present, Bi-LSTM and its derived models have been widely used in time series prediction scenarios^16^. In dealing with time series problems, introducing Self-Attention module in RNN based encoder–decoder model can overcome the “forgetting problem” of original model, and how to introduce self-attention into LSTM is a research hotspot in recent years. In the international communication short video dissemination model designed in this paper, BiLSTM model combined with Attention is used as the basic algorithm framework, and the hierarchical structure between different features is realized by residual link method.

## Methods

### Construct a system of key and objective indicators for international communication of short videos

This section introduces and explains the methodology used for constructing the key objective indicator system for international communication of short videos.

Common communication studies are often based on information dissemination sequences or social networks only, which is difficult to model complex information dissemination effectively. In recent years, more and more scholars have combined communication models based on the social network with neural networks and achieved good results^13^. However, short video communication differs from traditional communication in that it lacks social attributes and is more determined by short video content, platform recommendation algorithms, and user behavior. Therefore, this paper will combine short video content, platform recommendation algorithm, and user behavior to select key objective indicators, build indicator system, and then conduct subsequent research.

The interpretation of key objective indicators (KOIS) is divided into two parts, key and objective. One meaning of key indicators is to play a key role in the communication of short videos, and another meaning is to facilitate scholars to study the indicators qualitatively or quantitatively in the actual research process; objective indicators refer to the indicators that are not influenced by human subjective factors, such as specific numbers, for example, the number of plays and likes, and specific descriptions, such as Chinese and English. Under the above definition, combined with the actual operation, the following key objective indicators that need to be used in this paper are summarized, as shown in Table 1.

**Table 1:** Key objective indicators of short video dissemination.

Indicator type	Indicator name
Short video content indicators	Content
	Pattern
	Language
	Originality
User behavior indicators	Dig_count
	Comment_count
	Download_count
	Share_count
Communication indicators	Watch_count_speed
	Watch_count_acceleration
	Watch_count

After completing the selection of indicators, this paper continues to elaborate the hierarchical relationship of indicators. According to the dynamic and static properties of key objective indicators, the key objective indicators are horizontally divided into two categories of first-level indicators.

- *Static indicator* The key objective indicators that cannot be changed after the release of short videos, including content type, production mode, presentation language and originality.
- *Dynamic indicator* The key objective indicators that will change over time after the release of short videos, including likes, comments, downloads, shares, play growth rate, play growth acceleration and play volume. According to the cause and effect relationship of dynamic indicators, dynamic indicators are horizontally divided into two types of secondary indicators.
- *Status indicator* A dynamic indicator that does not evaluate the communication power of short videos directly.
- *Outcome indicator* A dynamic indicator that directly serve as the basis for evaluating the communication power of short videos, the play volume.

The reasons for using the play volume as an indicator to evaluate the communication power of short videos are as follows: A term similar to the concept of play volume is flow, which refers to the amount of exposure distributed by the servers to video. Play volume is the number of views. The behavior of the current audience on the short video platform is divided into two types, one is leisure and entertainment, and the other is shopping. Video play volume = Exposure * Exposure click rate; Likes = Exposure * Exposure click rate * Video like rate; Live turnover = Exposure * Exposure click rate * Viewing-commodity click rate * commodity click-order rate * customer unit price; In addition, in the communication of short videos, almost all quantifiable indicators such as comments, downloads, shares and so on can be broken down into similar formulas, and all of them contain exposure. Ideally, exposure should be used as the ultimate indicator for evaluating the spread of short videos, but as mentioned in the previous words, this paper only studies the front end data which are easy to collect. Play volume is the closest one to exposure, so it will be used as the basis for evaluating the spread of short videos, i.e. the Outcome Indicator.

**Figure 1:**
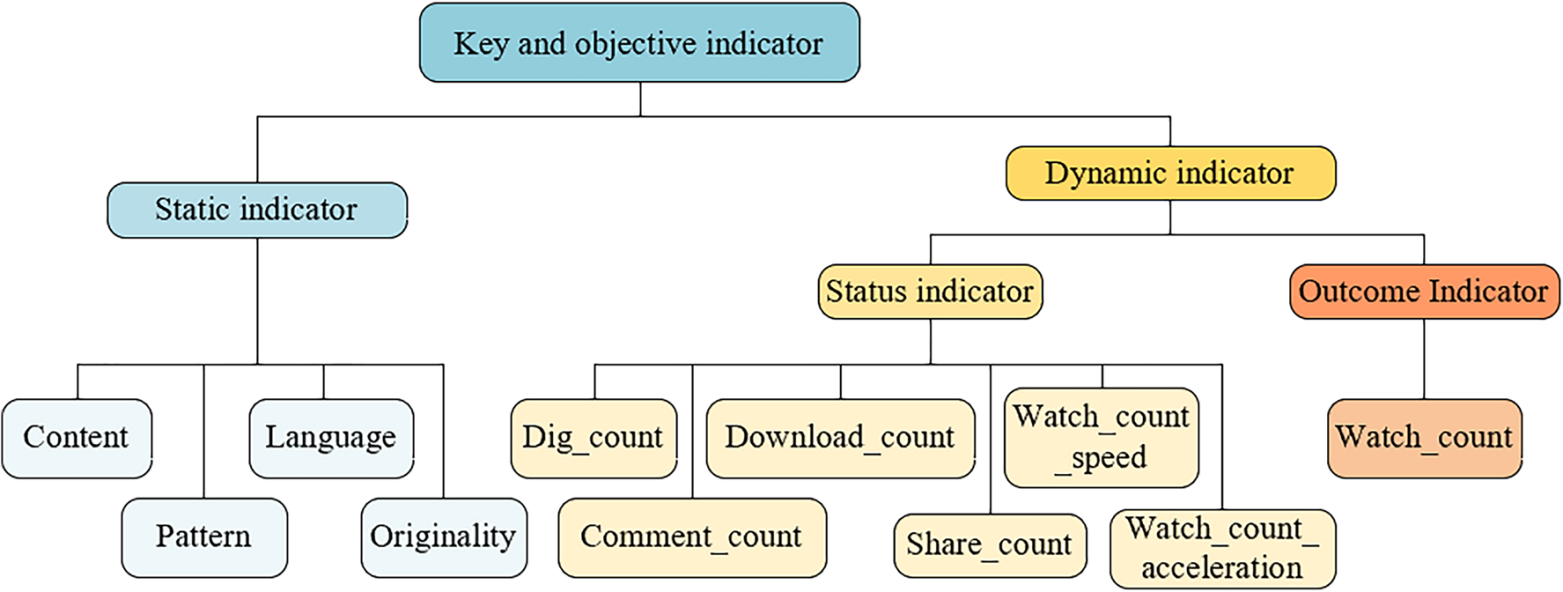
Key and objective indicators system of international communication short video.

According to the elaboration of the above hierarchical relationship, a set of key objective indicators system with hierarchy for short video communication is constructed, as shown in Fig. 1.

**Figure 2:**
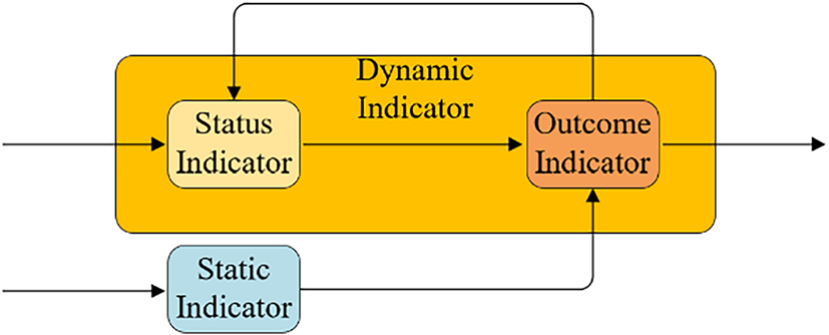
Short video communication network based on the key and objective indicators system of short video communication.

Combining the index system and the temporal characteristics of short video communication, this paper constructs a short video communication network based on the key and objective indicators system of short video communication (KOIS-PN). The network structure is shown in Fig. 2.

The network in the figure can be understood that the status indicators and the static indicators jointly determine the short video communication results. Considering the temporal characteristics of short video communication, the short video communication results in the current moment are fed back to the status indicators in the future. It is easy to see that the structure of the graph is very similar to the basic framework of recurrent neural network, therefore, this paper adopts the approach based on Attention and Bi-LSTM neural network for the international communication research of international communication short videos.

### AT-BiLSTM neural network model based on Attention and LSTM

**Figure 3:**
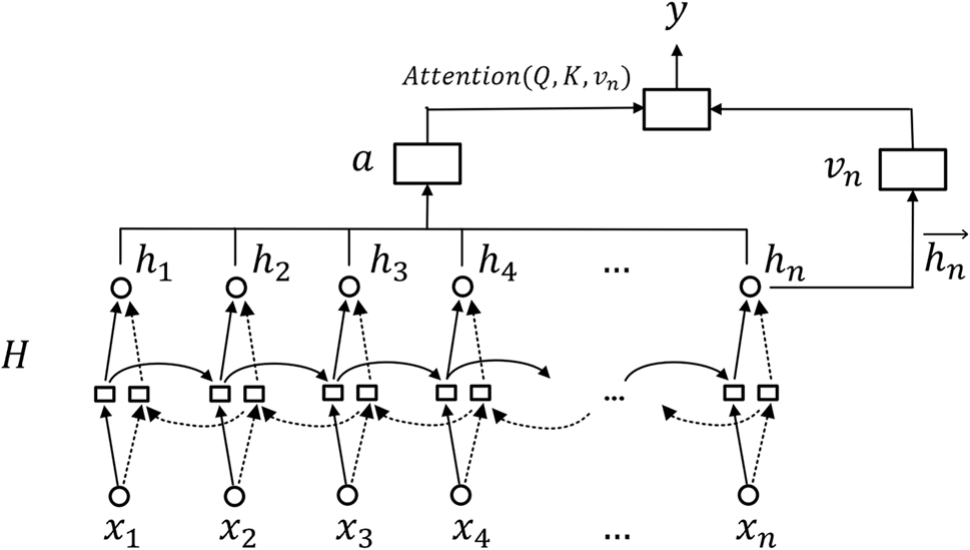
Structural framework of AT-BiLSTM neural network model.

The Attention-based Bi-LSTM model is actually the introduction of Attention into the Bi-LSTM framework. The structural framework designed in this paper is shown in Fig. 3.

Following Fig. 3, $x=({x}_{1}, {x}_{2}, \dots {x}_{n})$ is the input of the model. Bottom-up H from Fig. 3 is the Bi-LSTM neural network module. Solid lines from Fig. 3 denote forward LSTM layers and dashed lines from Fig. 3 denote reverse LSTM layers; in the output part of the Bi-LSTM neural network module, $\overrightarrow{{h}_{n}}$ from Fig. 3 denotes the final state value of the forward LSTM in H, and $a$ from Fig. 3 is the Attention probability of the information about all time positions for the final status of the forward LSTM, and $y$ from Fig. 3 is the final output. Since the experiments conducted in this paper are playback predictions, we only focus on that final status of the forward LSTM in the two-layer Bi-LSTM, i.e. $\overrightarrow{{h}_{n}}$. $Attention(Q,K,{v}_{n})$ which contains the $\overrightarrow{{h}_{n}}$ association information with the history moment information and is the history moment information at any position, which alleviates the long-distance forgetting problem of Bi-LSTM. The specific calculation process is shown in Eqs. (1)–(4):

1 $$H=\left[\begin{array}{c}\overrightarrow{H}\\ \overleftarrow{H}\end{array}\right]=({h}_{1}, {h}_{2}, \dots {h}_{n})$$

2 $$Q=H{W}^{Q},\; K=H{W}^{K},\; {v}_{n}=\overrightarrow{{h}_{n}}{W}^{v}$$

3 $$a=softmax\left(\frac{Q{K}^{T}}{\sqrt{{d}_{k}}}\right)$$

4 $$Attention\left(Q,K,{v}_{n}\right)=a{v}_{n}=softmax\left(\frac{Q{K}^{T}}{\sqrt{{d}_{k}}}\right){v}_{n}$$

$H$ denotes the input sequence $x=({x}_{1}, {x}_{2}, \dots {x}_{n})$ after Embedding by Bi-LSTM module. ${W}^{Q},{W}^{K},{W}^{v}$ are the weight matrices. $a$ contains ${h}_{1}, {h}_{2}, \dots {h}_{n}$, the information on the degree of similarity between different location. ${v}_{n}$ contains the final status information of the forward LSTM in the Bi-LSTM. $a$ with ${v}_{n}$, the weight sum of the two is obtained as $\overrightarrow{{h}_{n}}$ and ${h}_{1}, {h}_{2}, \dots {h}_{n}$. And $y$ is the final output.

### Short video communication model combining KOIS-PN and AT-BiLSTM

This section introduces and explains the methodology used for developing the international communication network model.

Among the key objective metrics of short videos, the model very easily ignores the static data due to the presence of static metrics, i.e., data that do not have temporal characteristics and are simply fed together into the time series model. Therefore, this paper will adopt a combination of the international communication short video communication network based on the short video communication key objective indicator system and the Attention-based Bi-LSTM model to study the international communication of short videos accordingly. The indicators in the system of Key and objective indicators constructed are specifically summarized into four categories:

- Category I: Content, Pattern, Language, Originality.
- Category II: Dig_count, Comment_count, Download_count, Share_count.
- Category III: Watch_count_speed, Watch_count_acceleration.
- Category IV: Watch_count.

**Figure 4:**
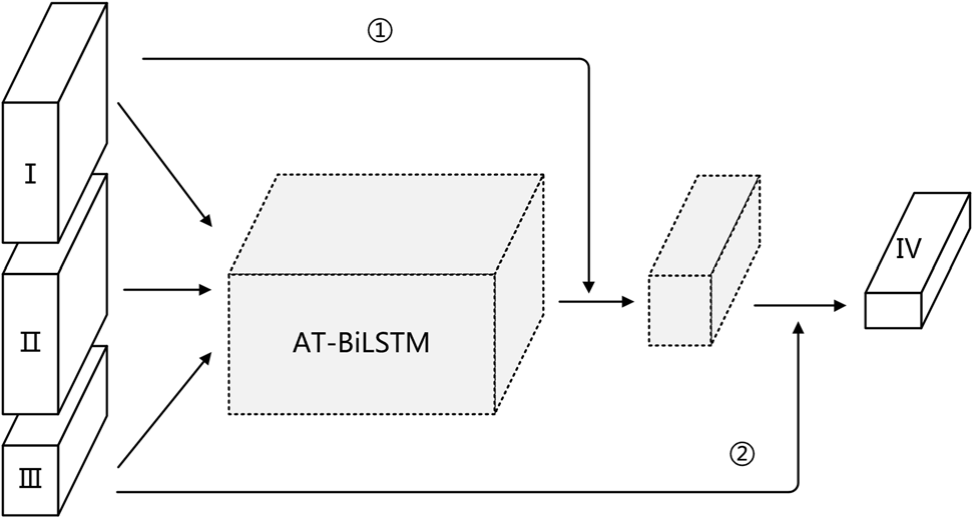
Base framework of the KOISPN-AT-BiLSTM model.

Combining the short video communication network in A and the AT-BiLSTM model in B, the underlying framework of the KOISPN-AT-BiLSTM model is shown in Fig. 4. As can be seen from Fig. 4, the structure of the short video communication network is represented by the residual structure and the time series model AT-BiLSTM. The residual structure is constructed along the following lines:

The residual structure ①, starting from the leftmost part of the frame diagram, all metrics except the predicted metric play are input into AT-BiLSTM at the same time. At this time, due to the lack of time characteristics of the first category, i.e. static indicators, two scenarios will occur after input into the model: One is that the AT-BiLSTM almost ignores the features of this category, and the other is that the AT-BiLSTM learns certain information and treats the static indicator I as a large bias. To prevent information loss, the first class of indicators are summed with the output of the AT-BiLSTM module by means of the residual connection ① and fed into the next layer of the neural network;

The residual structure ②, since the information contained in the category III indicators is the information of the forecast indicator on the growth acceleration and rate, which are closely related to velocity in the physical concept. Therefore, it is directly input to the decision level of the model by means of residual connections ②.The residual structure ① addresses the problem of forgetting non-time series in the time series model, and the residual structure ② addresses the problem of allowing a smoother flow of information about the growth of the forecast results.

**Figure 5:**
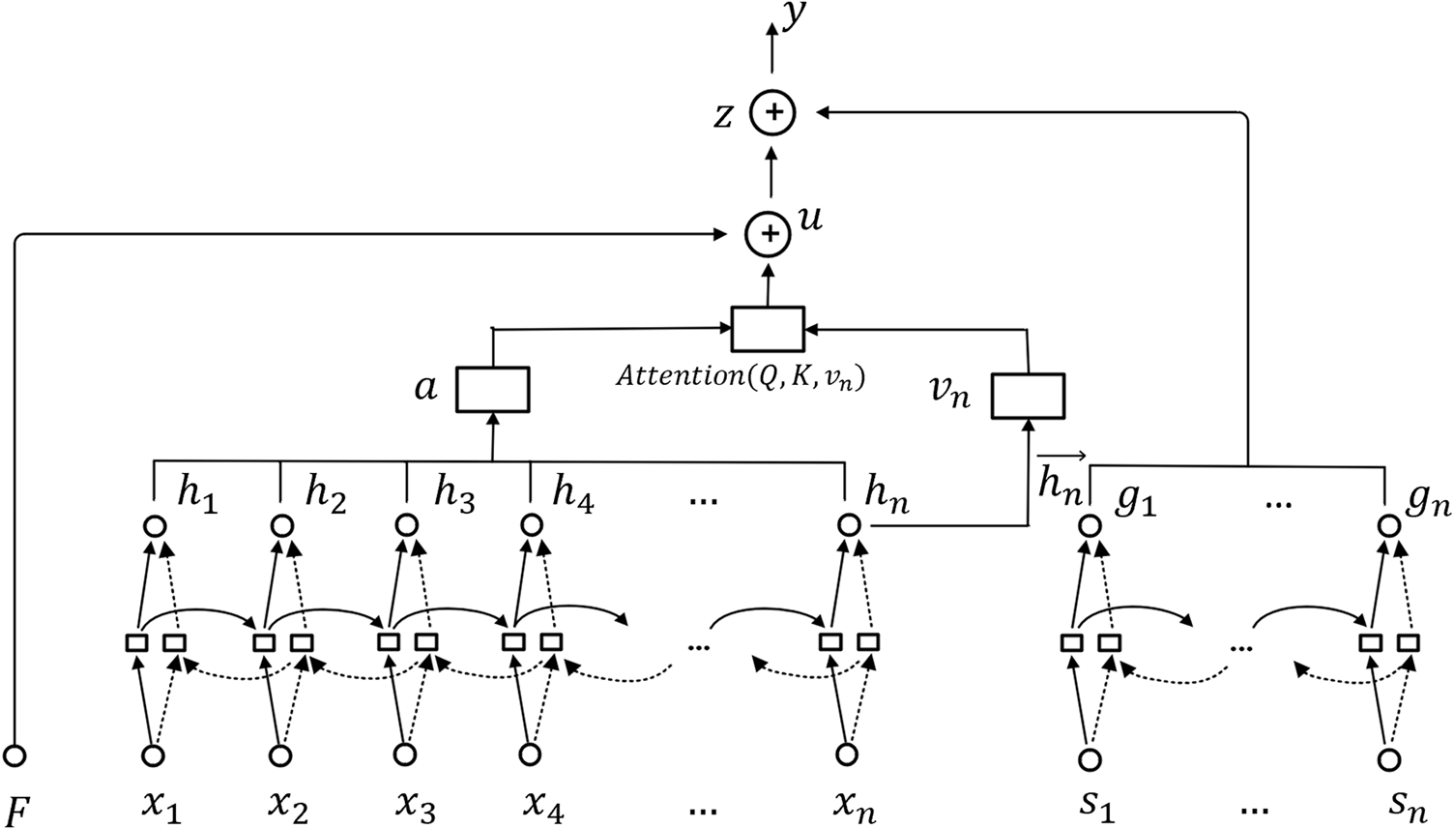
KOISPN-AT-BiLSTM model structure.

Expanding the basic framework of the model yields the specific model structure shown as Fig. 5.

The index of category I is denoted as $F$, and at the moment $t$, the input of the AT-BiLSTM module is denoted as ${x}_{t}$. ${x}_{t}$ which contains all the representations except the predictors. The third category of indicators at the moment $n$ is denoted as ${s}_{n}$. ${h}_{i}$ is the output in the layer of the Bi-LSTM at the moment $i$. $\overrightarrow{{h}_{t}}$ is the final status in the layer of the forward LSTM at the moment $t$. $y$ is the prediction result. The complete forward algorithm is as follows:

(i) Calculation $${H}_{t},{G}_{t}$$

The output of the forward hidden layer of the Bi-LSTM is denoted as $\overrightarrow{{h}_{t}}$, and the output of the inverse hidden layer is denoted as $\overleftarrow{{h}_{t}}$. The hidden status of the network in this layer is denoted as ${H}_{t}$, which is calculated as shown in Eqs. (5)–(12):

5 $$\overrightarrow{{h}_{t}}=\overrightarrow{LSTM}({h}_{t-1},{x}_{t},{c}_{t-1})$$

6 $$\overleftarrow{{h}_{t}}=\overleftarrow{LSTM}({h}_{t+1},{x}_{t},{c}_{t+1})$$

7 $${H}_{t}=\left[\begin{array}{c}\overrightarrow{{h}_{t}}\\ \overleftarrow{{h}_{t}}\end{array}\right]$$

8 $${i}_{t}=\sigma ({W}_{xi}{x}_{t}+{W}_{hi}{h}_{t-1}+{b}_{i})$$

9 $${f}_{t}=\sigma ({W}_{xf}{x}_{t}+{W}_{hf}{h}_{t-1}+{b}_{f})$$

10 $$c_{t} = f_{t} \odot c_{t - 1} + i_{t} \odot tanh\left( {W_{xc} x_{t} + W_{hc} h_{t - 1} + b_{c} } \right)$$

11 $${o}_{t}=\sigma ({W}_{xo}{x}_{t}+{W}_{ho}{h}_{t-1}+{b}_{o})$$

12 $$h_{t} = o_{t} \odot tanh\left( {c_{t} } \right)$$

$\sigma (x) =1/ (1+exp (-x) )$, $tanh$ is the hyperbolic tangent function, ${i}_{t}$ is the input gate, ${f}_{t}$ is the forgetting gate, ${o}_{t}$ is the output gate, ${c}_{t}$ is the cell status, $W$ and $b$ denote the weight matrix and bias vector of the network, $\odot$ denotes the corresponding element multiplication.

(ii) Calculation $$Attention\left(Q,K,{v}_{n}\right)$$

13 $$H=\left[\begin{array}{c}\overrightarrow{H}\\ \overleftarrow{H}\end{array}\right]=({h}_{1}, {h}_{2}, \dots {h}_{n})$$

14 $$Q=H{W}^{Q},K=H{W}^{K},{v}_{n}=\overrightarrow{{h}_{n}}{W}^{v}$$

15 $$a=softmax\left(\frac{Q{K}^{T}}{\sqrt{{d}_{k}}}\right)$$

16 $$Attention\left(Q,K,{v}_{n}\right)=a{v}_{n}=softmax\left(\frac{Q{K}^{T}}{\sqrt{{d}_{k}}}\right){v}_{n}$$

$H$ denotes the input sequence $x=({x}_{1}, {x}_{2}, \dots {x}_{n})$ after two layers of Bi-LSTM module Embedding. ${W}^{Q},{W}^{K},{W}^{v}$ are the weight matrices. $a$ contains the similarity information between different locations of ${h}_{1}, {h}_{2}, \dots {h}_{n}$. ${v}_{n}$ contains the final status of the forward LSTM in the Bi-LSTM. The weight sum of $a$ and ${v}_{n}$ is obtained as the connection between $\overrightarrow{{h}_{n}}$ and ${h}_{1}, {h}_{2}, \dots {h}_{n}$, $Attention\left(Q,K,{v}_{n}\right)$.

(iii) Calculation output $y$

17 $$u=softmax(F{W}^{fu}+Attention\left(Q,K,{v}_{n}\right){W}^{at-u})$$

18 $$z=softmax(u{W}^{uz}+G{W}^{gz})$$

19 $$y=1/ (1+exp (-z{W}^{zy}) )$$

This model has several features relative to the usual time series forecasting models:

One is that the model framework is constructed based on the communication network. Previous algorithms in the middle of the neural network were interpreted so poorly that a deeper network had to be designed to solve the problem. The architecture involved in this paper is highly explanatory, and there are few models that combine metric networks with neural networks in the research field of short videos. Besides, based on this architecture, the model addresses the problem of forgetting non-time series in the time series model and the problem of smoother information flow by means of residual connection.

The other is that this paper combines the specific situation of short video communication and designs a network that incorporates the Attention mechanism into Bi-LSTM, so that after the input is Encode Embedding, the algorithm can learn the association between the final status and the arbitrary previous location, which overcomes the long-distance forgetting problem of Bi-LSTM. For example, if a short video with a very good trend suddenly stops playing, it is more likely that the short video has entered the background audit to suspend the distribution of traffic. In this case, the model should refer more to the information from more distant locations, in which case the module actually serves to filter out the anomalous information and enhance the robustness of the model.

## Experiment

This section introduces and explains the specific techniques, algorithms, and data sources employed.

The coefficient of determination^17^, also known as the goodness of fit, is expressed as ${R}^{2}$. The coefficient of determination reflects what percentage of the fluctuations in the dependent variable *y* can be described by the fluctuations in *x*. That is what percentage of the representation variation in the dependent variable *y* can be explained by the independent variable* x*.

The definition is shown in Eqs. (20)–(23):

20 $${R}^{2}=1-\frac{{SS}_{res}}{{SS}_{tot}}$$

21 $${SS}_{tot}=\sum_{i=1}^{n}{\left({y}_{i}-\overline{{y }_{i}}\right)}^{2}$$

22 $${SS}_{res}=\sum_{i=1}^{n}{\left({y}_{i}-{f}_{i}\right)}^{2}$$

23 $$\overline{{y }_{i}}=\frac{1}{n}\sum_{i=1}^{n}{y}_{i}$$

In the equation, the total error is ${SS}_{tot}$, the random error is ${SS}_{res}$, ${y}_{i}$ is the predicted value, $\overline{{y }_{i}}$ is the mean predicted value, ${f}_{i}$ is the true value, n is the number of samples. From the formula, it can be seen that the total error ${SS}_{tot}$ is caused for two reasons: the first one is the size of the output sequence length n and the second one is the random error caused by the degree of model merit. Obviously, the smaller the random error is, the larger the ${R}^{2}$ is, the closer it is to 1. ${R}^{2}$ is often used for performance testing of regression models. In general, the model can be considered as a good fit when ${R}^{2}>0.8$. In this paper, ${R}^{2}$ is used as the evaluation algorithm of the model.

### Dataset construction

In this paper, we selected 38 TikTok creators with recent and stable output, whose videos are all related to China and the content types are Chinese food or Chinese teaching. According to the short video communication key objective indicator system, the data to be crawled are like rate, comment rate, download rate, share rate, play growth rate, play growth acceleration and play volume. Starting from the release of the video, we crawled its likes, comments, downloads, shares and plays every 1 h. A total of 435,962 data and 2150 videos were crawled, with a size of about 80 M. The experimental data were collected in the time range of 2022.05.01–2022.8.17, and the specific data descriptions are shown in Table 2.

**Table 2:** Data description.

Fields	Meaning
ID	Custom record ID
USERNAME	User name
UID	User ID corresponding to user name
DESC	Video description
CDN	Video address
Dig_count	Number of likes
Comment_count	Number of comments
Watch_count	Number of video plays
Down_count	Number of video downloads
Share_time	Number of video shares
Create_time	Video creation time, timestamp format
Aid	Video ID (unique)
Video_url	Video address
Date	Date of observation
Hour	Observation time point
From_unixtime	Video creation time, datetime format

### Experimental analysis

This section introduces and analyzes model performance, providing the strengths, limitations, and potential areas for improvement of the KOISPN-ATBiLSTM model.

The experiments of Bi-LSTM-based short video broadcast prediction are divided into two groups. Group A is based on a single Bi-LSTM; Group B is based on the Bi-LSTM model and incorporates the Key and Objective Indicator System Based Communication Network (KOIS-PN) constructed in this paper.

**Figure 6:**
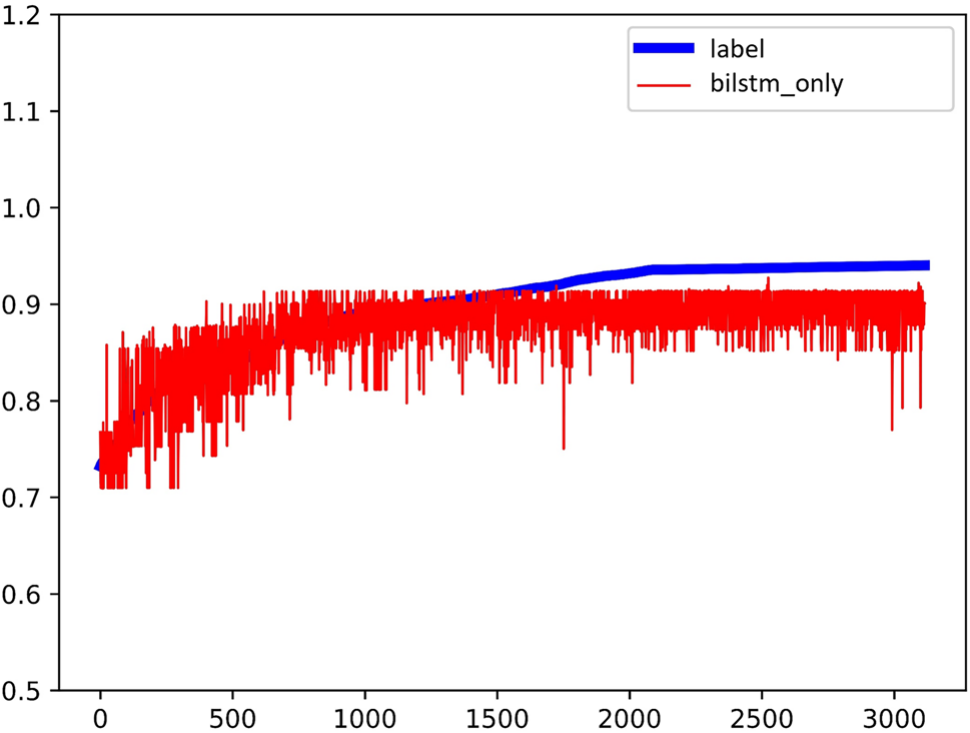
Experimental results of Bi-LSTM.

**Figure 7:**
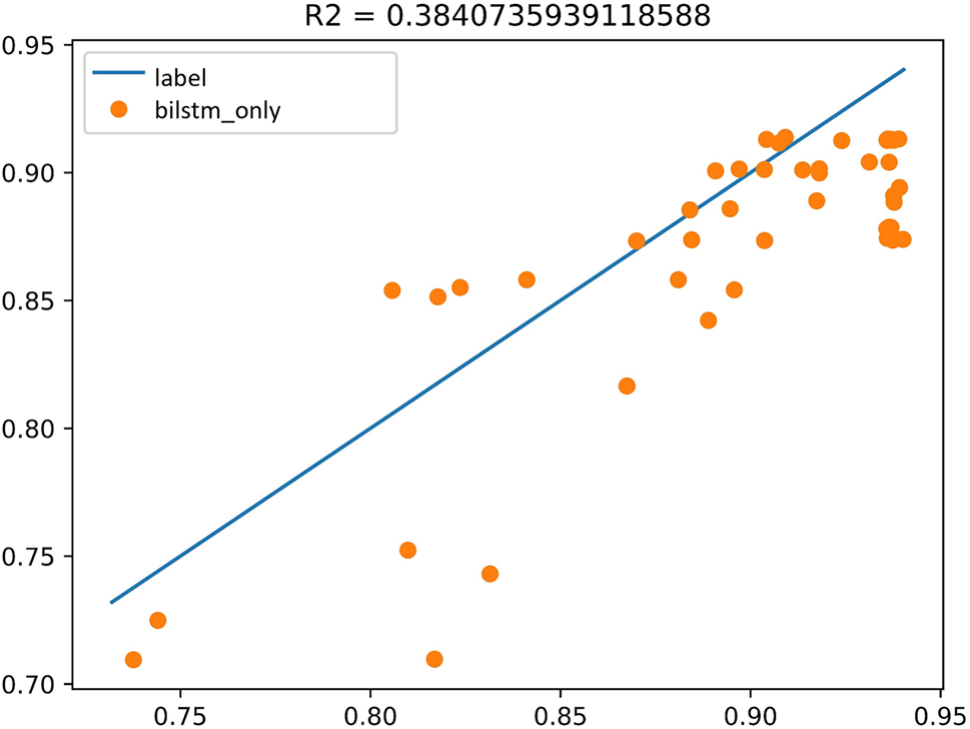
The coefficient of determination of the experimental results of Bi-LSTM.

Experiment A takes all the Key and objective indicators except the outcome-playback as the input sequence and playback as the output sequence label. Label is the blue line drawn in the Fig. 6. The prediction result of Bi-LSTM is the red line drawn in the Fig. 6. The better the fit, the blue line and the red line are about close to a line; in the Fig. 7, the horizontal axis x is the true value, the vertical axis y is the predicted value, the orange dot indicates the value of y in the case that the true value is x, and the blue line indicates the predicted result of fitting to a straight line. The closer the blue line is to the diagonal y = x, the better the experimental result is. The right graph is more intuitive than the left graph. The coefficient of determination of the experimental results is ${R}^{2}=0.38$.

**Figure 8:**
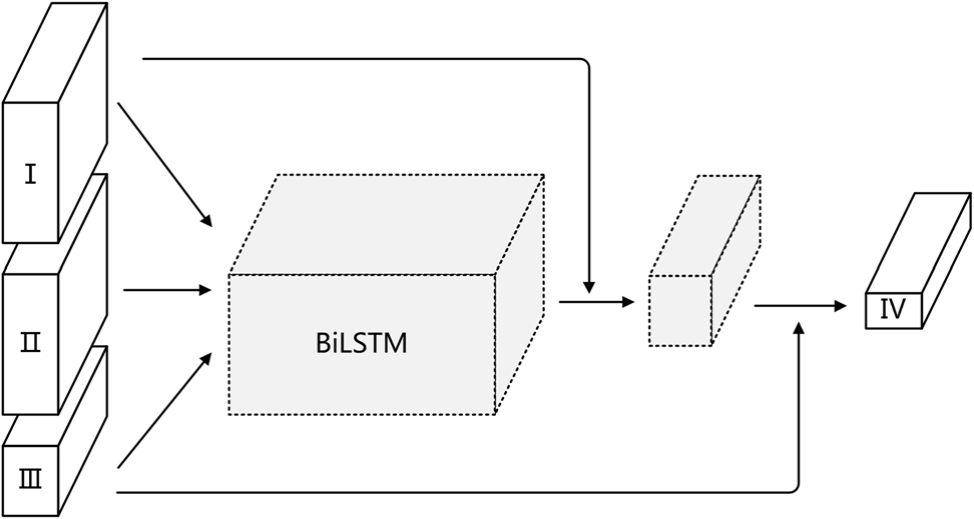
Structure of KOISPN-BiLSTM model.

Experiment B fuses the short video communication network based on the key objective metric system with Bi-LSTM. The principle is the same as the communication model that combines the communication network based on the key objective indicator system with the Attention-based Bi-LSTM neural network model. All 11 Key and objective indicators are divided into four groups: the first group is static indicators with content type, production mode, presentation language, and originality; the second group is the number of likes, comments, shares, and downloads; the third group is the growth rate of play volume, the acceleration of play volume growth; the fourth group is play volume. The indicators in the first, second and third groups are input to the input layer of the model independently. The first and third sets of indicators are directly connected to the decision level through residual connections, and meet with the transformation results of the first three sets of indicators in the decision layer to jointly generate outputs. The structure is shown in Fig. 8.

**Figure 9:**
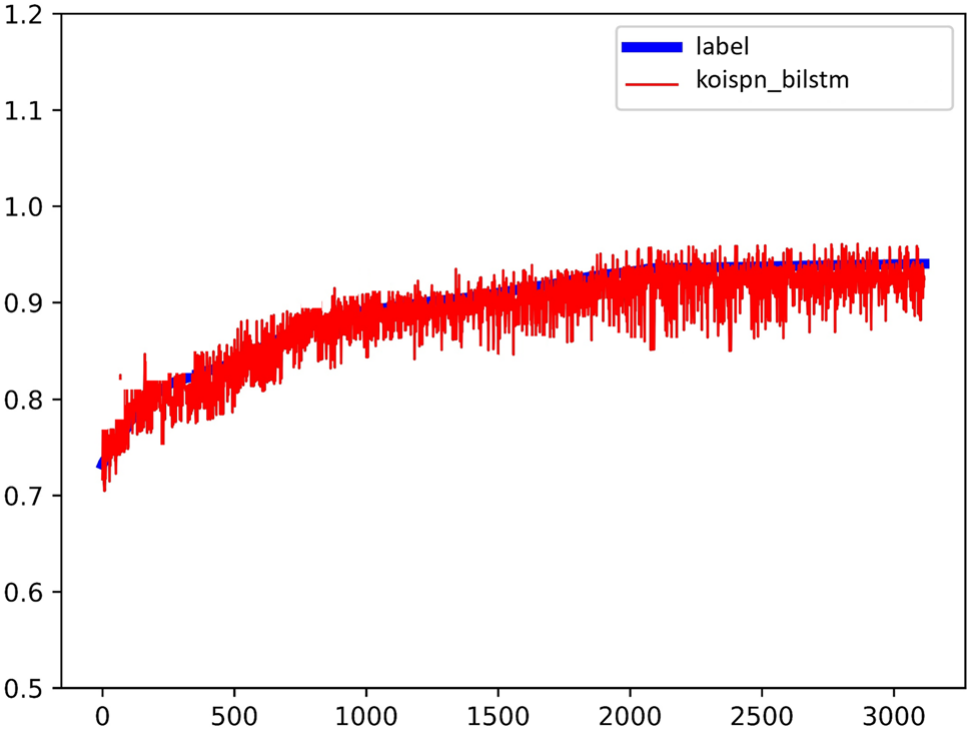
Experimental results of KOISPN-BiLSTM.

**Figure 10:**
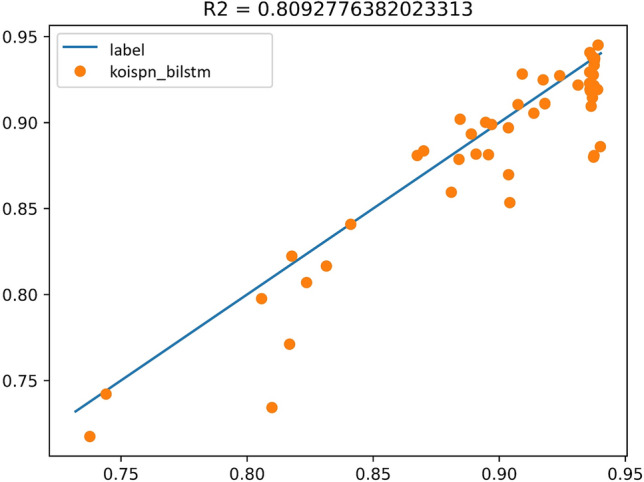
The coefficient of determination of the experimental results of KOISPN-BiLSTM.

The predictions of the KOISPN-BiLSTM model are shown in Figs. 9 and 10, with Label as the blue line and the prediction as the red line plotted in the Fig. 9. The experimental result coefficient of determination is ${R}^{2}=0.81$.

It can be seen that the BiLSTM model incorporating the short video communication network based on the key objective indicator system significantly outperforms the effect of the single BiLSTM model.

The experiments of Attention-based short video playback prediction are divided into two control groups. Group C is an experiment based on a single Multi-Head-Attention model, while Group D is an experiment incorporating the KOISPN-Attention model based on the Key and Objective Indicator System Based Communication Network (KOIS-PN) constructed in this paper.

**Figure 11:**
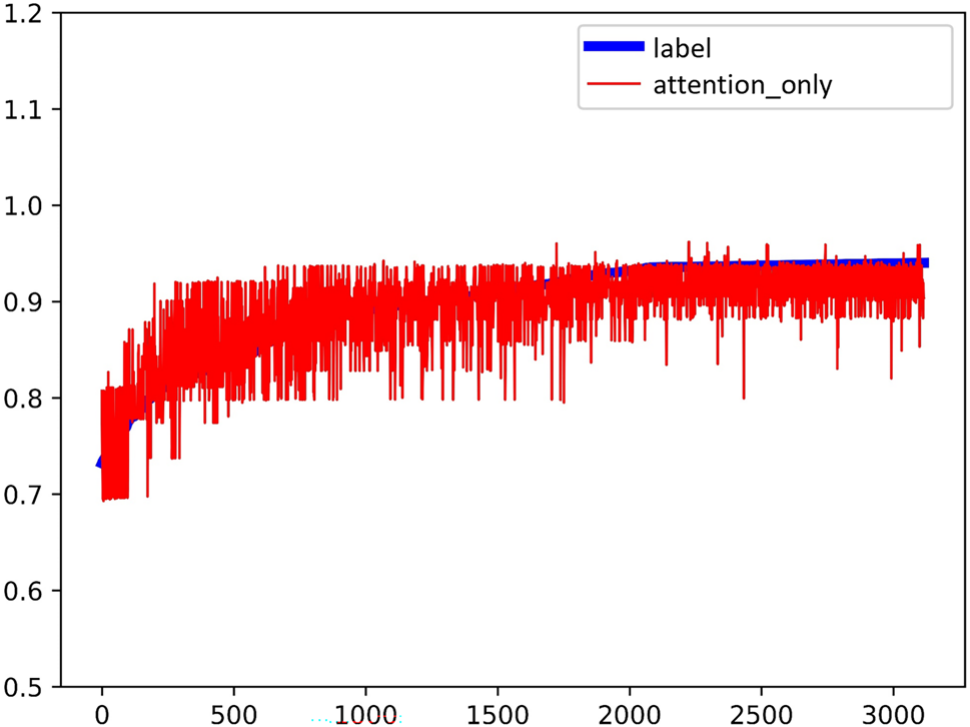
Experimental results of Attention.

**Figure 12:**
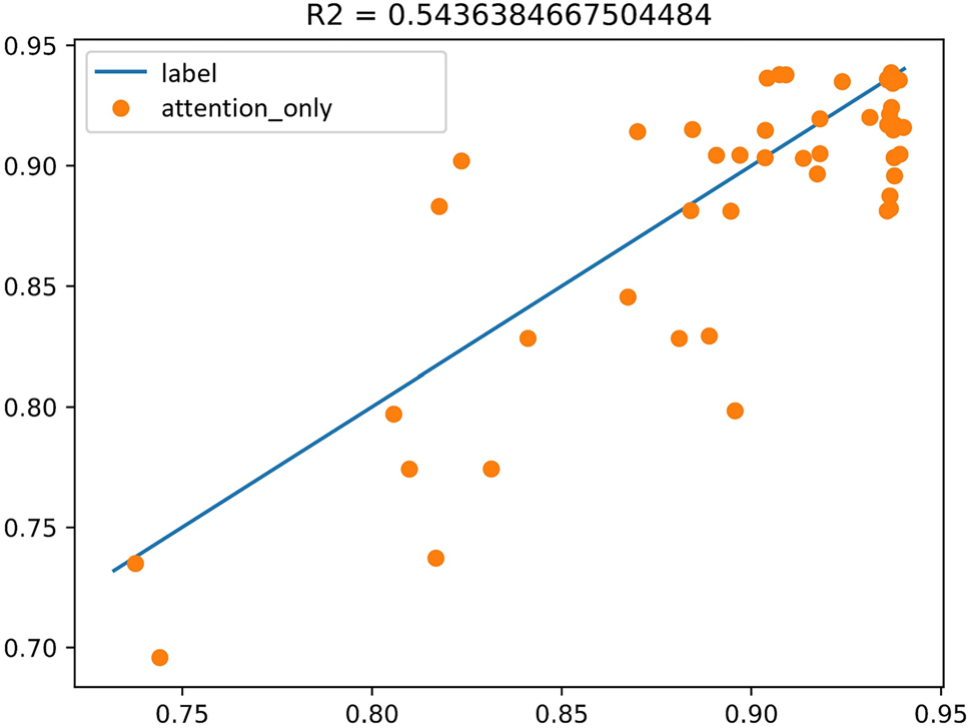
The coefficient of determination of the experimental results of Attention.

Experiment C takes all of the key and objective indicators except the outcome indicator-play volume as the input sequence, and the play volume as the output sequence label. Label is the blue line plotted in the Fig. 11, and the predicted result of the Multi-Head-Attention model is the red line plotted in the Fig. 11. The experimental result coefficient of determination is ${R}^{2}=0.54$ (Fig. 12).

**Figure 13:**
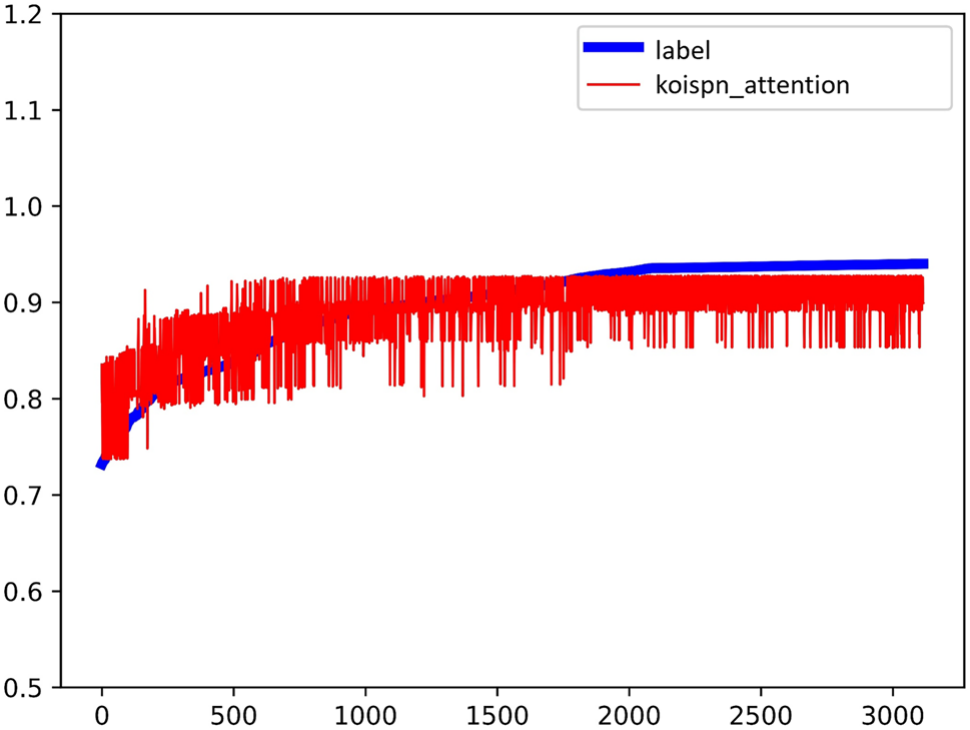
Experimental results of KOISPN-attention.

**Figure 14:**
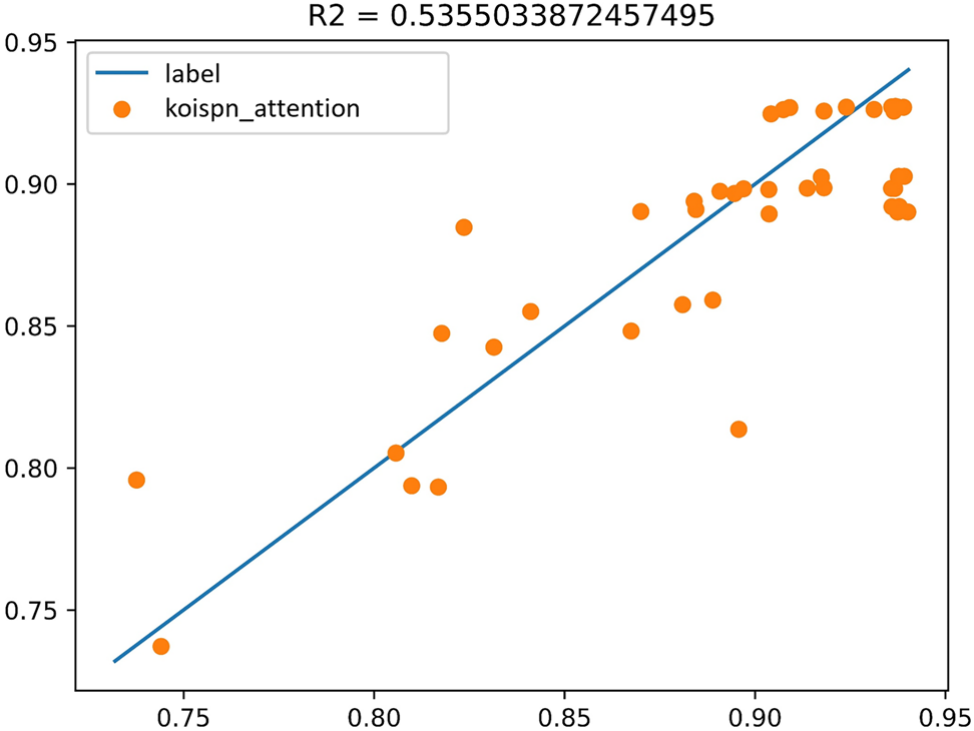
The coefficient of determination of the experimental results of KOISPN-attention.

Experiment D integrates the short video communication network based on the key objective indicator system in Multi-Head-Attention, the same as Fig. 13. The experimental results are shown in Fig. 14 and the coefficient of determination is ${R}^{2}=0.53$.

The above figure shows that the Attention model of KOISPN, which incorporates a short video communication network based on the key objective indicator system, is comparable to the prediction effect of a single Attention model.

The experiments of AT-BiLSTM-based short video playback prediction are divided into two control groups. Group E is an experiment based on a single AT-BiLSTM model and Group F is an experiment incorporating the KOISPN-AT-BiLSTM model based on the Key and Objective Indicator System Based Communication Network (KOIS-PN) constructed in this paper.

**Figure 15:**
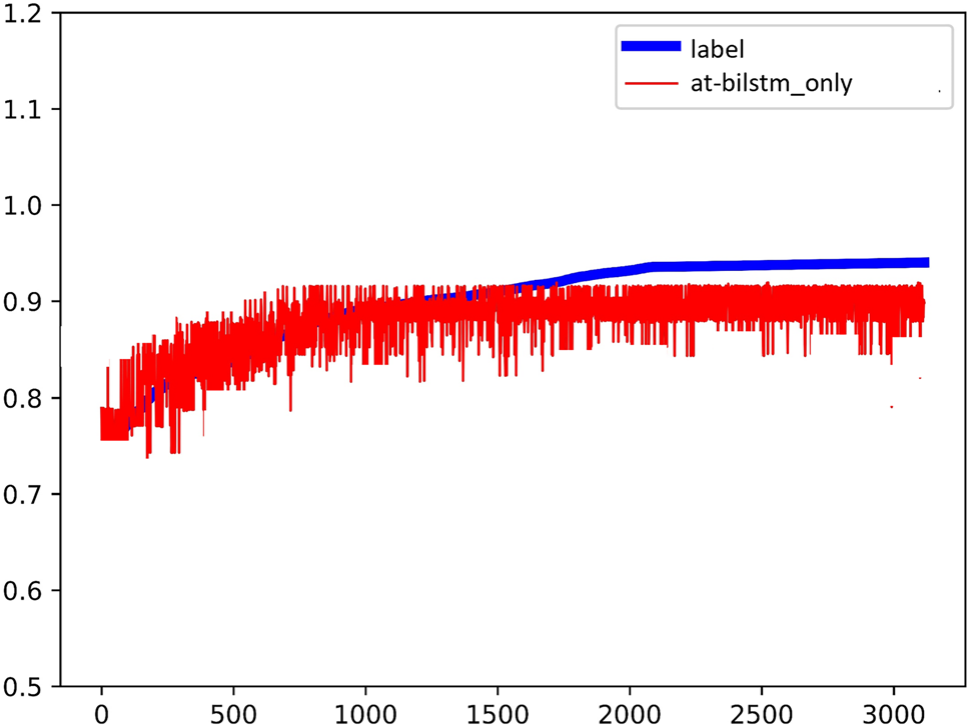
Experimental results of AT-BiLSTM.

**Figure 16:**
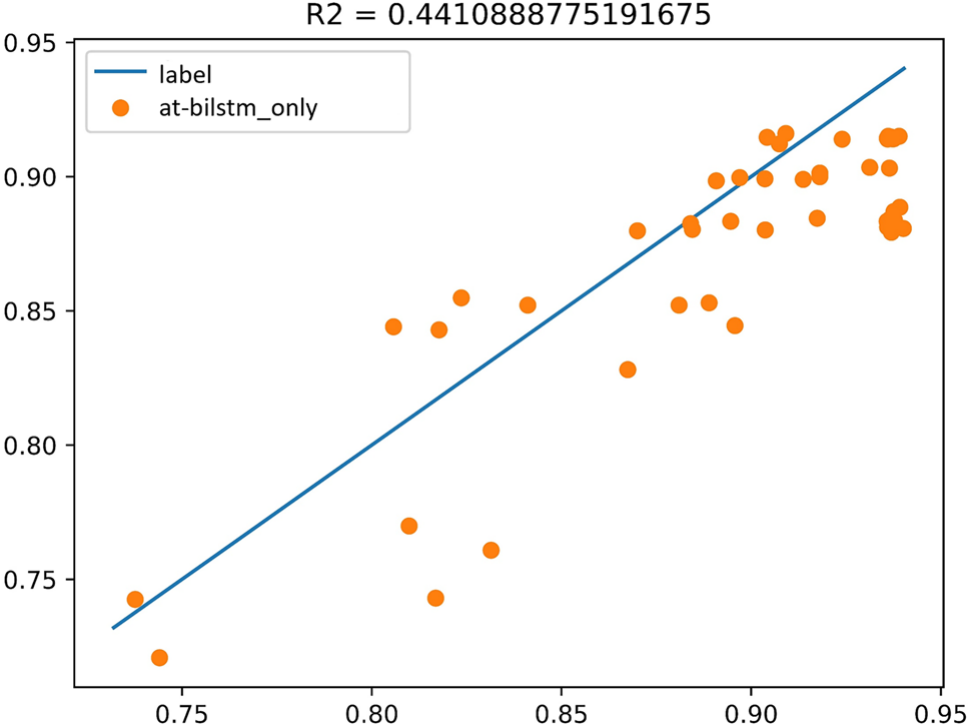
The coefficient of determination of the experimental results of AT-BiLSTM.

The model for Group E experiments is similar to Fig. 15, and the experimental results are shown in Fig. 16, ${R}^{2}=0.44$.

**Figure 17:**
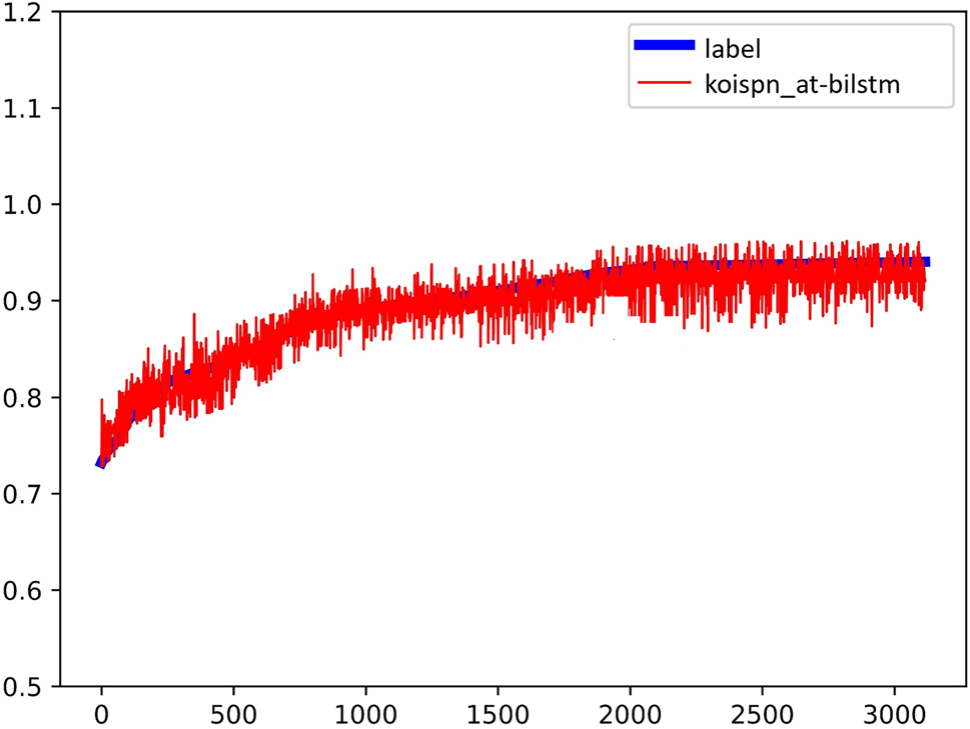
Experimental results of KOISPN-AT-BiLSTM.

**Figure 18:**
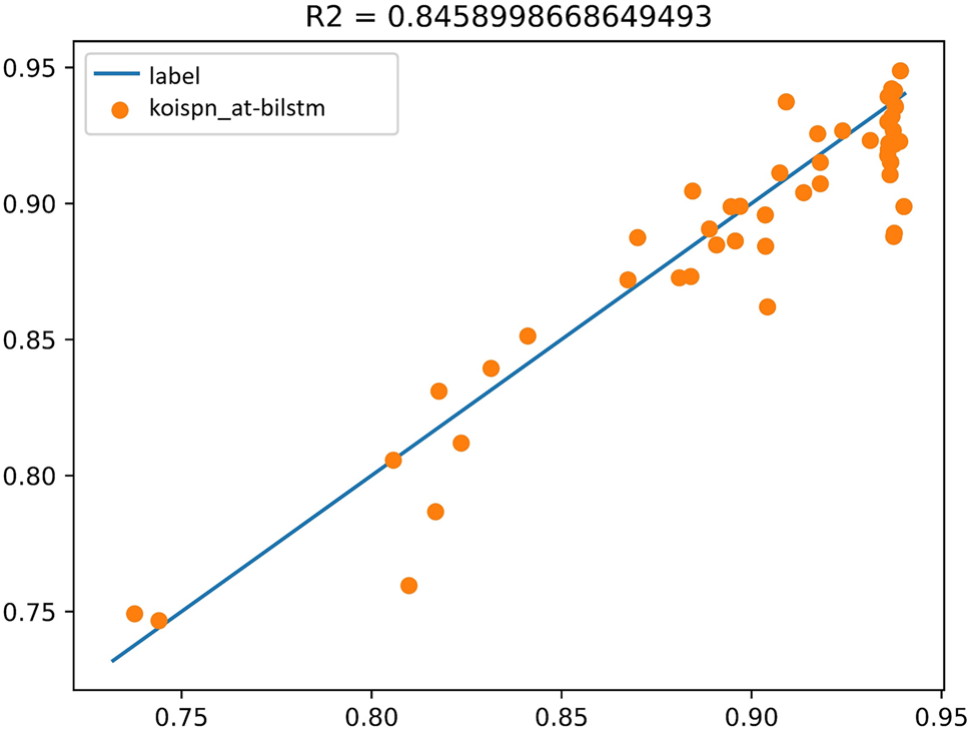
The coefficient of determination of the experimental results of KOISPN-AT-BiLSTM.

The model for Group F experiment is shown as Fig. 17, and the experimental results are shown in Fig. 18, ${R}^{2}=0.84$. As seen in the above figure, the AT-BiLSTM model KOISPN-AT-BiLSTM, which incorporates the short video communication network based on the key objective indicator system, significantly outperforms the effect of the single AT-BiLSTM model.

## Results

**Figure 19:**
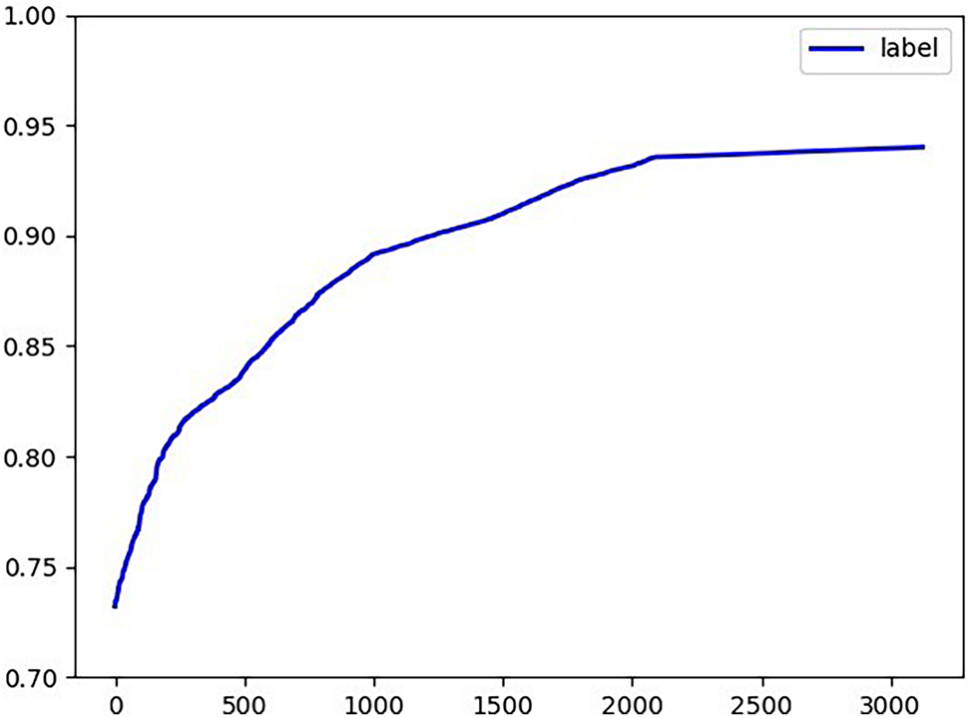
True value of short video plays.

In order to be able to show the experimental results clearly, the real value of short video plays is presented separately as shown in Fig. 19.

We can summarize the following growth characteristics of play volume based on the growth curve of play volume:

- The play volume curve is monotonically increasing;
- The growth of play volume is non-linear;
- The growth of play volume is mainly concentrated in the period when the video is first released, and then the growth gradually slows down and gradually stabilizes.

**Figure 20:**
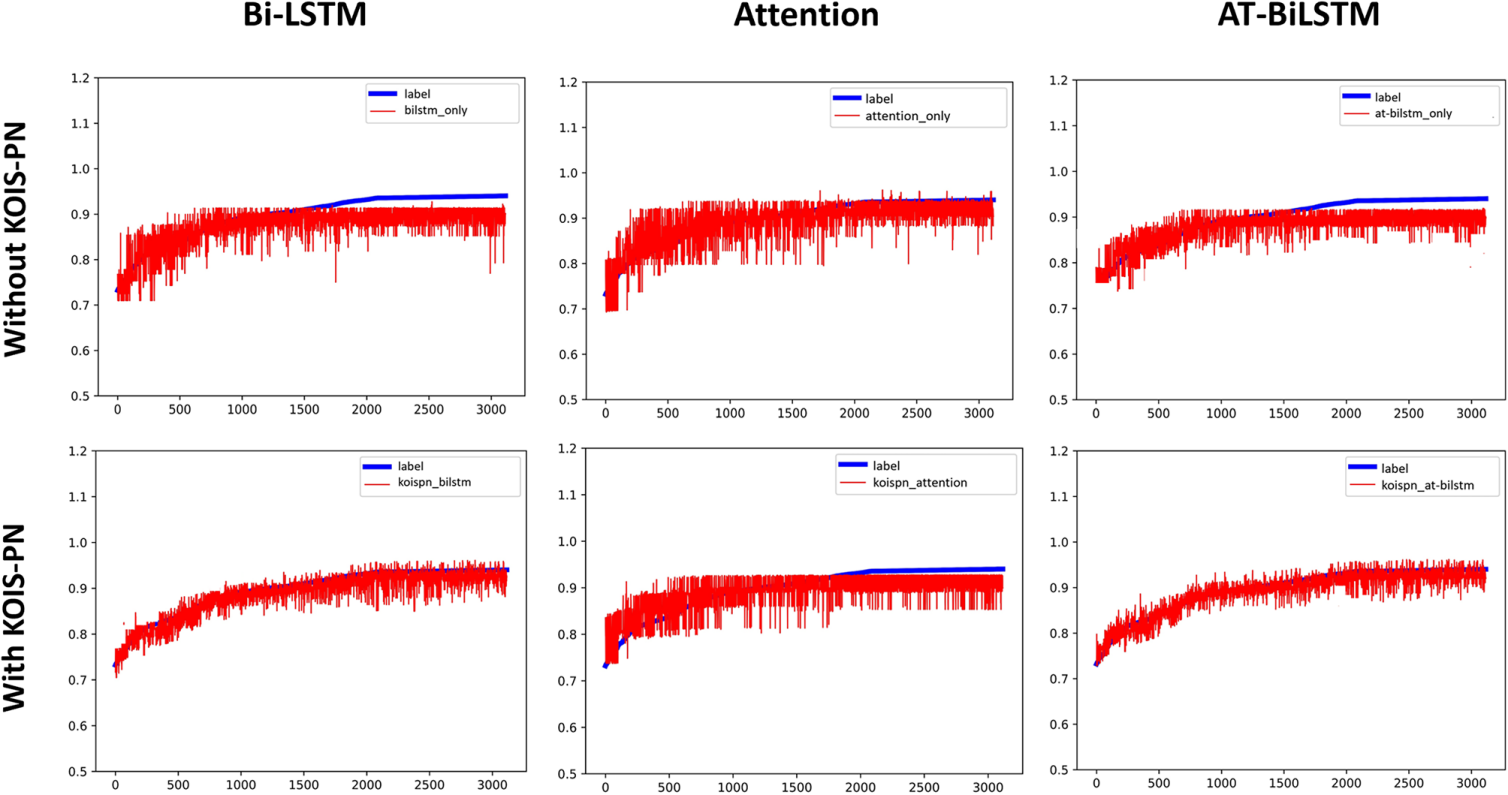
Comparison of the experimental results of six groups of models (I).

**Figure 21:**
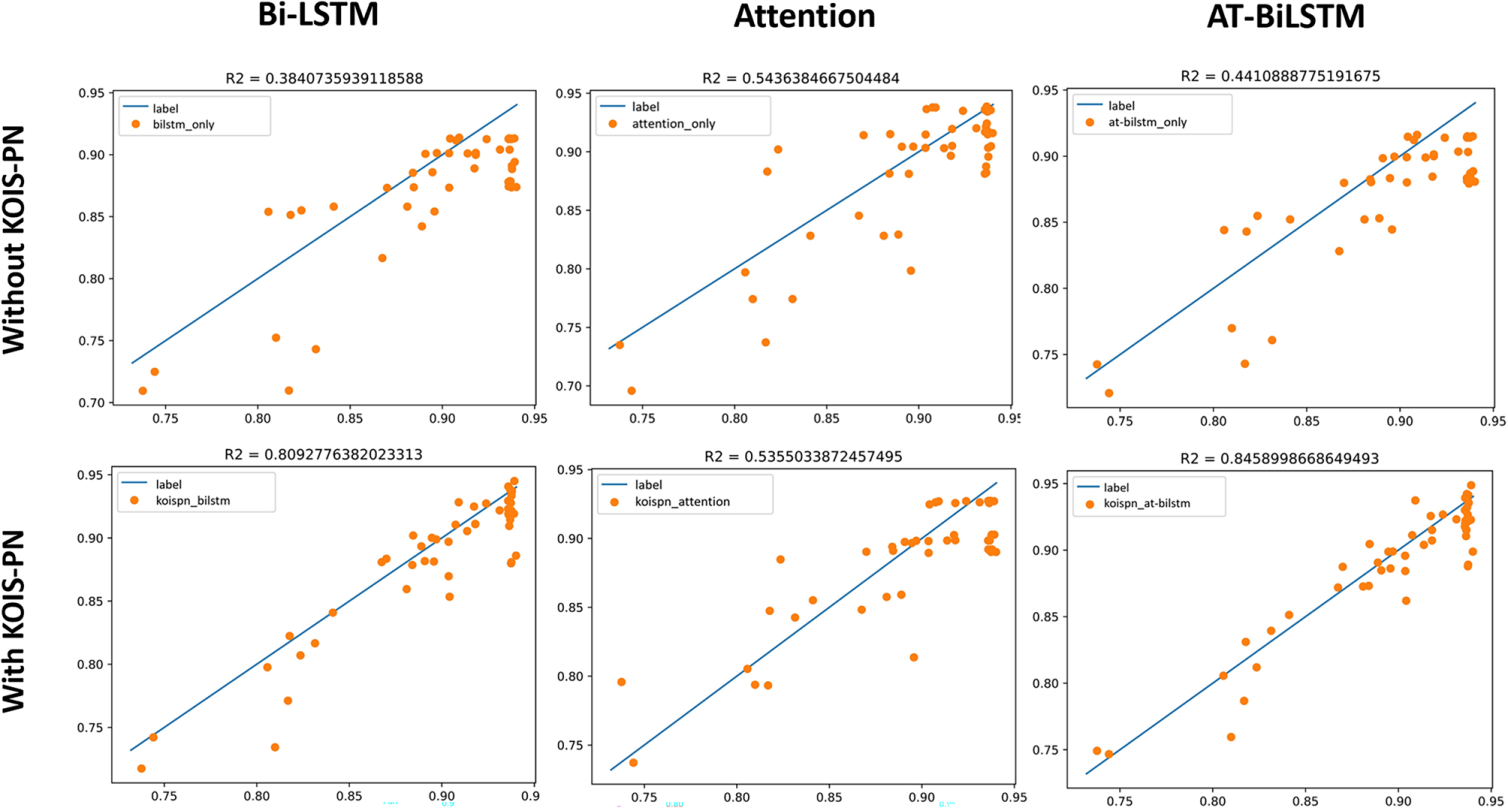
Comparison of the experimental results of six groups of models (II).

The comparison of the experimental results of different methods is shown in Figs. 20 and 21.

The comparison table of the results of ${R}^{2}$ is shown in Table3:

(i) For the short video communication model with Bi-LSTM as the base framework, the performance is improved by about 113% by introducing KOIS-PN, a short video communication network based on the key objective metric system. The reason for the performance improvement analyzed in this paper is that KOIS-PN solves the applicability problem when the conventional LSTM model applied directly to short videos. For the conventional Bi-LSTM model, all features are equally entered into the model at once, regardless of the attributes of the features. For example, static indicator data as a kind of information that does not change with time, it is obviously not reasonable enough to input it directly into the Bi-LSTM model that deals with time series and the experimental results also prove this idea that ${R}^{2}$ is only 0.38. The introduction of KOIS-PN is equivalent to treating the information of different attributes independently. One is to prevent the single Bi-LSTM from losing non-time information, where the static indicator can be interpreted as a large bias; the other is to connect the play growth rate and the play growth acceleration, which are intuitively important metrics for decision making, so that the information can be delivered more smoothly. Overall, the introduction of KOIS-PN is actually for matching the model structure with the data characteristics.
(ii) For the Attention-based neural network short-video communication model, after introducing KOIS-PN, a short-video communication network based on the key objective indicator system, the model performance is comparable to that of the unintroduced model. The results of this experiment show that a single Attention model does not exactly match the problem studied in this paper so this study will improve and optimize the model on the basis of Bi-LSTM.
(iii) The performance of the short video communication model with AT-BiLSTM as the base framework is improved by about 91% after introducing KOIS-PN, a short video communication network based on the key objective indicator system. The analysis is similar to that in (i).
(iv) The KOISPN-AT-BiLSTM model performs better than the KOISPN-BiLSTM model. The introduction of Attention makes the performance of LSTM-based models improve by about 3.7%, we analyze that this is because the Attention module can notice the information that cannot be learned by BiLSTM as well as the models incorporating KOISPN. For example, in Chapter II we discussed that there is a large amount of human intervention in the recommendation mechanism of short videos. If the video playback growth is suspended due to the entry audit, the conventional model may assume that the future playback of the video tends to be flat. This is actually the long-range forgetting problem of LSTM, and the introduction of Attention can alleviate this problem.

**Table 3:** Comparison of results.

Model	Bi-LSTM	Attention	AT-BiLSTM
Without KOIS-PN	0.38	0.54	0.44
With KOIS-PN	0.81	0.53	0.84

## Conclusion

Firstly, this paper constructs the international communication short video key objective indicator system, introduces the definition, selection and design logic of Key and objective indicators, and the idea about constructing the corresponding indicator system, and then constructs the international communication short video key objective indicator system and related data set from this. Secondly, this paper constructs the international short video communication network model. The construction of the international short video communication network is divided into three steps: The first step is to construct KOIS-PN, a short video communication network based on the Key and objective indicators system; the second step is to construct the AT-BiLSTM neural network model; the third step is to construct the international short video communication network model combining KOIS-PN and AT-BiLSTM; the final step is to validate the model performance. The experimental results illustrate that the core theoretical model proposed in this paper, KOISPN-ATBiLSTM, has certain validity and advancement.

The KOISPN-ATBiLSTM model proposed in this paper can not only realize the broadcast prediction of short videos, but also describe the mapping relationship from the Key and objective indicators of short videos' communication to the international communication effect. If we can specifically study the relationship between some of these indicators and the results, the model can be used to provide creation suggestions for the short videos with different types and different needs, so that it can be applied to the field of short video communication research.

## Data Availability

All data included in this study are available upon request by contact with the corresponding author.
